# Experiences in Coping with Stress—A Qualitative Study of Family Caregivers of Children with Medical Complexity

**DOI:** 10.3390/children11091151

**Published:** 2024-09-23

**Authors:** Mikhaila N. Layshock, Amy S. Porter, Jori F. Bogetz, Lydia McLachlan, Sydney Weill, Abby Rosenberg, Joseph G. Winger, Amy Houtrow, Robert B. Noll, Yael Schenker, Justin A. Yu

**Affiliations:** 1 University of Pittsburgh School of Medicine, 3550 Terrace Street, Pittsburgh, PA 15213, USA; 2 Division of Supportive and Palliative Care, Department of Pediatrics, Mass General for Children, Massachusetts General Hospital, Harvard Medical School, Boston, MA 02114, USA; abporter@mgh.harvard.edu; 3 Division of Bioethics and Palliative Care, Department of Pediatrics, Treuman Katz Center for Pediatric Bioethics and Palliative Care, Center for Clinical and Translational Research, Seattle Children’s Research Institute, University of Washington School of Medicine, Seattle, WA 98195, USA; jori.bogetz@seattlechildrens.org; 4 Case Western Reserve University School of Medicine, Cleveland, OH 44106, USA; lmm271@case.edu; 5 Department of Internal Medicine, University of Pittsburgh Medical Center, Pittsburgh, PA 15213, USA; sydney.weill@ynhh.org; 6 Division of Psychosocial Oncology and Palliative Care, Dana Farber Cancer Institute, Department of Pediatrics, Boston Children’s Hospital, Harvard Medical School, Boston, MA 02114, USA; abbyr_rosenberg@dfci.harvard.edu; 7 Department of Psychiatry and Behavioral Sciences, Duke University School of Medicine, Durham, NC 27710, USA; joseph.winger@duke.edu; 8 Department of Physical Medicine and Rehabilitation, University of Pittsburgh School of Medicine, Pittsburgh, PA 15213, USA; houtrow@upmc.edu; 9 Department of Psychology, University of Pittsburgh, Pittsburgh, PA 15213, USA; rbn1@pitt.edu; 10 Division of General Internal Medicine, Section of Palliative Care and Medical Ethics, Palliative Research Center (PaRC), University of Pittsburgh School of Medicine, Pittsburgh, PA 15213, USA; yas28@pitt.edu; 11 Division of Pediatric Supportive and Palliative Care, University of Pittsburgh School of Medicine, UPMC Children’s Hospital of Pittsburgh, Pittsburgh, PA 15213, USA; yuja@upmc.edu

**Keywords:** children with medical complexity, family caregivers, emotional well-being, coping strategies, psychosocial needs

## Abstract

Objective: To better understand the strategies family caregivers of children with medical complexity (CMC) utilize to deal with the stress and challenges associated with caregiving. Methods: We conducted a cross-sectional qualitative study among family caregivers of CMC receiving medical care at a children’s hospital in Western Pennsylvania. Participants completed in-depth, semi-structured interviews focused on how CMC family caregivers approach and manage caregiving-related challenges and stress. Using constant comparative methodology, we inductively analyzed deidentified transcripts for emergent themes. Results: We interviewed 19 participants (89.4% female) with a mean age of 43 years (range 32–54 years). The mean age of the participants’ children was 10.8 years (range 1–20 years). Twelve participants’ children identified as white and four identified as Black. Three central themes regarding CMC caregivers’ stress-coping strategies emerged: (1) maintaining a positive mindset, (2) developing and relying on interpersonal support networks, and (3) making time for self-preservation. All three themes were universally reported (*n* = 19/19) by our participants. The most common subthemes for each theme, respectively, focused on staying hopeful and celebrating moments of joy; cultivating supportive relationships with family, friends, and fellow CMC family caregivers; and finding pleasure in “little things” (e.g., everyday activities and hobbies). Conclusion: Family caregivers of CMC utilize a multi-faceted approach to cope with the stress and challenges routinely encountered in caring for CMC. This study’s findings could be used to inform future clinical efforts and research directions aiming to improve clinicians’ ability to support CMC caregivers’ well-being.

## 1. Introduction

Children with medical complexity (CMC) live with chronic health conditions, major functional limitations (e.g., difficulty with feeding and cognition), and health service needs such as home-nursing. CMC also often require medical technologies (e.g., wheelchairs, mechanical ventilation) to sustain life and daily function [1]. The CMC population has roughly doubled over the past decade (1.6% of the US pediatric population [1.2 million US children]) and account for approximately one-third of all pediatric health care expenditures and one-half of inpatient pediatric-related costs [2]. Most CMC live at home, with family caregivers responsible for arranging and carrying out intricate care plans, which include demanding medical tasks, and coordinating communication between providers. CMC caregivers also encounter challenges such as fragmented health care systems, financial adversity, inadequate home-based services, and complicated medical decisions [3,4]. These challenges put CMC caregivers at high-risk for reduced emotional well-being [5]. CMC caregivers have reported reduced overall mental health and health-related quality of life, high levels of emotional distress, and poor sleep-related health [5].

Reduced emotional well-being among CMC family caregivers can hinder their ability to care for and advocate for their children [6,7]. In other parental caregiver populations, emotional distress, sleep loss, family conflict, and economic insecurity have been inversely linked to child health [6]. For example, Goodwin et al. demonstrated that an increased volume of stressors (i.e., family conflict) was associated with more hospitalizations among children with asthma [8]. This has serious public health implications, as CMC caregivers play an essential role in the operational capacities of pediatric health systems [9,10].

In response, experts and national advocacy organizations have specifically called for targeted efforts aiming to improve CMC family caregiver emotional well-being [11,12,13]. Currently, however, few psychosocial interventions have been developed for or evaluated among this caregiver population. A 2019 scoping review examining interventions aiming to improve the well-being of parents of children with special health care needs (a broader pediatric population in which CMC are often categorized) only identified one study that likely included CMC caregivers—a non-experimental pilot trial of dyadic peer support intervention [14,15]. Similarly, Edelstein et al.’s scoping review of approaches targeting CMC caregiver stress noted the overall lack of experimental data and that existing interventions predominately focused on enhanced models of care coordination and service delivery [16]. Consequently, clinicians lack knowledge of and training in evidence-based strategies to better support CMC family caregivers.

One promising approach would be to enhance CMC caregivers’ ability to cope with challenges perceived as stressful. In the broader pediatric caregiving literature, psychosocial interventions targeting family caregivers’ stress coping ability have improved caregiver emotional well-being [17,18]. For example, studies examining the effects of psychosocial interventions on caregivers of children with cancer have demonstrated that enhancing caregivers’ problem-solving skills can significantly decrease caregivers’ negative affectivity and post-traumatic stress symptoms when compared to usual psychosocial care [19,20,21].

However, directly applying existing psychosocial interventions to CMC family caregivers is not appropriate, as their unique experience is characterized by life-long challenges and frequent and demanding interactions with pediatric health systems. To successfully identify and tailor psychosocial interventions for CMC caregivers, a better understanding of the coping strategies (i.e., thoughts, behaviors, and resources) they use to deal with challenges and maintain their emotional well-being is needed. Therefore, to inform future selection and adaptation of existing psychosocial interventions, and to identify supportive practices that CMC clinicians can adopt now, we conducted qualitative interviews exploring CMC caregivers’ perspectives on managing stressful challenges related to their caregiving experiences.

## 2. Methods

This inductive phenomenological qualitative study was part of a larger study examining the experiences of family caregivers of CMC in coping with caregiving-related stress. We utilized the consolidated criteria for reporting qualitative research guidelines for conducting rigorous qualitative research [22]. The Institutional Review Board at the University of Pittsburgh approved all aspects of this study.

### 2.1. Participants

The participants (*n* = 19, Table 1) were family caregivers of CMC receiving medical care at a single quaternary academic pediatric hospital located in Western Pennsylvania. We used our institution’s Complex Care Center’s patient eligibility criteria to define medical complexity: chronic health conditions in 3 or more organ systems requiring subspecialist care and/or utilization of medical technology. Caregivers were eligible if they were able to complete interviews in English, had medical decision-making authority for their child, and were of ages ≥18 years. We excluded family caregivers of CMC who (1) resided in long-term care facilities, as these caregivers were not responsible for providing the majority of their child’s care, and (2) caregivers of children <1 year of age to ensure that participants had significant experience in caring for their child at home. To maximize the breadth of experiences, we enrolled one caregiver per family unit.

Eligible caregivers were identified through bi-weekly reviews of our hospital’s (1) Complex Care Center’s outpatient schedule, and (2) Palliative and Supportive Care team’s inpatient consult census. To minimize power differentials, we excluded caregivers whose child was receiving direct clinical care from the interviewer (JY) at the time of study enrollment. Prior to approach, caregiver eligibility was confirmed with the relevant team’s providers and permission to approach in-person and provide study details was obtained. All participants provided written informed consent prior to participating and were compensated USD 35. Caregivers were purposively sampled to attain diversity in socioeconomic status, children’s medical conditions, and time since initial diagnosis.

### 2.2. Instruments

The interview guide was developed by a group with expertise in palliative care (JY, YS), pediatric oncology (AR), pediatric rehabilitation medicine (AH), and clinical psychology (RN). The interview guide was refined based on feedback from three pilot interviews with representative CMC family caregivers to improve question clarity and acceptability. We used open-ended, non-leading questions to identify caregivers’ personal experiences and contributors to their stress experience, strategies for and struggles in coping with stressors, and beliefs about the impact of caregiving on their emotional well-being. Private, one-on-one, semi-structured interviews were conducted either in person or via telephone, based on the participant’s preference, by a single investigator (JY) with training in pediatrics, palliative care, and clinical research. Participant information was deidentified and interviews were recorded and transcribed verbatim with all identifying information removed. The data were only handled by members of the team directly associated with their coding. Interviews were conducted until the research team agreed that thematic saturation (when no new themes emerged from subsequent interviews) was achieved. Interview transcripts were uploaded to ATLAS.ti Web, a cloud-based qualitative analytics platform that facilitated data storage, organization, coding, annotation, and content analysis from our body of unstructured interview data.

### 2.3. Procedures and Data Analysis

Two study team members (ML and JY) developed a preliminary codebook iteratively and inductively through line-by-line coding and constant comparative methodology on an initial subset of transcripts. The codebook was finalized through multiple rounds of concept and code review and revision with the senior multidisciplinary team (YS, AR, AH, and RN). Four research team members (ML, JY, LM, and SW) then independently applied the final codebook to all transcripts using recursive cycles of coding and face-to-face meetings (during which disagreements were resolved through discussion and consensus). To enhance the credibility and clarity of our thematic analysis, we presented our findings to members of a community advisory board of CMC family caregivers. This advisory board provided feedback on which themes resonated and those which required refinement. Lastly, the coded interview data were organized into overarching themes by the entire study team through group discussion, iterative thematic analysis, and feedback from members of a community advisory board of CMC family caregivers. The proportions of participants reporting each theme and subthemes were determined to describe prevalence.

## 3. Results

We interviewed 19 CMC family caregivers between March 2022 and April 2023 (Table 1). The majority of participants identified as female (*n* = 17) and reported living with another adult (*n* = 13) and receiving home nursing services (*n* = 11) for an average of 42.5 h per week (SD 17.6 h, range 16–80 h). The average age of the caregivers was 43 years old (SD 6.7, range 32–54 years). The average age of the participants’ children was 10.8 years old (SD 6.3, range 1–20 years). Ten of the children were female (52.6%). Twelve (63.2%) identified as white, four (21.1%) identified as Black, and three (15.8%) identified as another race (e.g., Asian, mixed race). Nearly all interviews were conducted via telephone (18 of 19). The average length of the interviews was 49 min (range 37–66 min).

**Figure 1:**
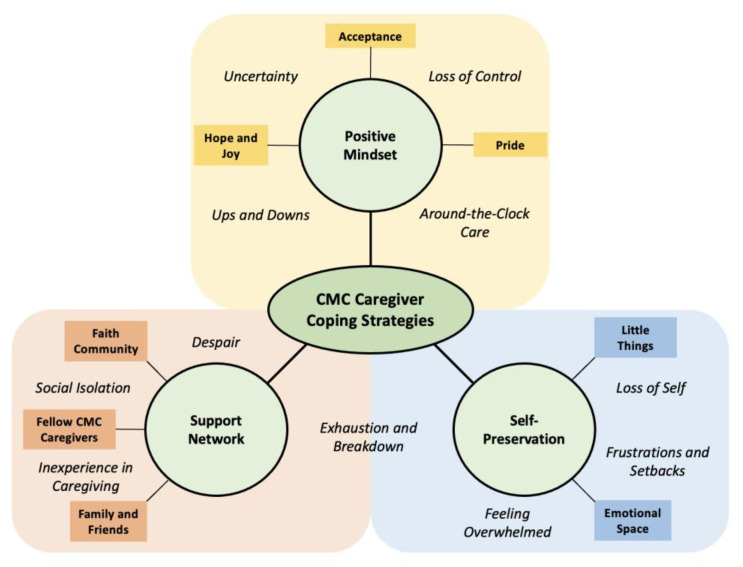
Conceptual model of the coping strategies employed by CMC family caregivers. This figure presents the coping strategies CMC family caregivers use to address the stress and challenges (italicized) they commonly experience.

Three overarching themes emerged in participants’ discussions about how they dealt with caregiving-related challenges and maintained their emotional well-being: (1) maintaining a positive mindset, (2) developing and relying on interpersonal support networks, and (3) making time for self-preservation. For each theme, we discuss the subthemes that describe the thoughts, behaviors, and resources which enable CMC caregivers to employ these approaches. Representative quotes for the themes and subthemes are presented in Table 2, Table 3 and Table 4. Our conceptual model (Figure 1) illustrates the assortment of coping strategies participants reported using to manage caregiving challenges.

### 3.1. Theme 1: Maintain a Positive Mindset

All participants (*n* = 19/19) described employing multiple ways of deliberate thinking and self-talk strategies to stay positive throughout caregiving’s rollercoaster experience (Table 2). Caregivers repeatedly explained that a positive mindset mitigated the intensity of negative emotions, which was critical for maintaining their long-term ability to endure the ups-and-downs of caregiving and ensuring their child’s health. The following subthemes describe concepts and strategies that participants relied on during times of acute stress and daily routines.

Hope and Joy (*n* = 18/19): Nearly all participants emphasized the importance of being hopeful about the future and seeking out joy in their lives. Discussions about hope typically centered on their child’s long-term health and/or their family’s overall circumstances. One mother reflected on her goals, “I’m hopeful that with [our son] being more mobile and interactive, and maybe being able to communicate, that the kids can play together. It will be more like a normal family”. Importantly, caregivers added that hope was dynamic, fluctuating in what they were hopeful for and how difficult it could be to hold on to. Multiple participants discussed experiences in which their hope was solely focused on getting through the challenge in front of them (e.g., admission to intensive care) and relying on their child’s history as a source of inspiration.

Intertwined with discussions about hope were caregivers’ concerted efforts to recognize and celebrate moments of joy. Caregivers explained that “small wins” in their child’s health or development were quite meaningful and helped them maintain a positive mindset. As one mother remarked, “I’d say probably the best thing is to admire and appreciate the strength of kids, how determined they can be… We’re excited over stuff that normal parents [couldn’t] care less to even recognize… We’re so lucky to get to see those moments”. Another participant described the impact of her daughter’s new wheelchair, “She will decide she doesn’t want to go certain places… she has more independence to say, ‘Yeah, look. I’m not going there. Just her being herself… she makes my heart smile”.

Acceptance (*n* = 14/19): Comments regarding hope for the future were often balanced by discussions about the importance of and their path to accepting the implications of their child’s health conditions. Acceptance enhanced caregivers’ ability to reconcile with uncertainty and regulate intense emotions. Central to acceptance was the understanding that, at times, aspects of their child’s care and well-being were out of their control and intentionally not dwelling on issues they could not change. One father summarized:

It is what it is. Nothing can be done or changed, so I kind of accept it… I just learned to not really stress over stuff that you can’t change, because stressing over something you can’t change doesn’t do anything. It just wears you out. You’ve got to accept it and keep going on.

Pride (*n* = 12/19): Many participants discussed the sense of pride that stemmed from how their care enabled their children’s flourishing. One mother stated, “The decisions I make always revolve around her, and I always feel like they have her best interest in mind… I feel like I’m good and I’m decent”. Caregivers also experienced deep fulfillment from ensuring their child felt loved. Participants also used this concept as a protective mechanism in the face of acute stress (e.g., child illness) and found solace knowing they were caring for their child as best as possible. One participant summarized the self-talk she uses to deal with setbacks, “Give yourself grace because you are doing the best that you can and giving her absolutely everything… [you don’t need] to feel that tremendous guilt”.

### 3.2. Theme 2: Developing and Relying on an Interpersonal Support Network

Every participant (*n* = 19/19) discussed the importance of cultivating a network of support beyond their child’s health care team (Table 3). Caregivers derived emotional support, encouragement, caregiving assistance, and practical advice from these relationships. Participants also discussed how their support network provided a safe place to vent about frustrations and other stressors.

Family and Friends (*n* = 17/19): Family and friends offered both practical and emotional support, which helped alleviate caregiving-related stress. This included respite and/or an “extra set of hands”, helping problem-solve in the moment (e.g., malfunctioning feeding pump), companionship, and moral support during times of acute stress. Emphasizing the value of emotional support, one participant stated, “I couldn’t imagine going through this alone… if I’m feeling down, he’ll lift me up. It’s almost like we take turns. It’s like a blessing that both of us are in that dark space together at the same time”.

In The Trenches (*n* = 17/19): Participants highlighted the importance of developing relationships with people who could relate to the challenges of CMC caregiving. Fellow caregivers were a trusted source for practical advice (e.g., wheelchair adjustments) and discussing concerns about their child’s medical care plan. For example, one mother described her relationship with an aunt whose child also lives with neurologic conditions, “She has a daughter that [has] autism and epilepsy, and so I can talk to [her] about seizures, [seizure] meds, developmental delays, and the worries that go along with all those things”. Participants also valued the camaraderie they felt with other CMC caregivers, which helped alleviate feelings of social isolation stemming from the difficulty in relating to parents of children without complex needs who “don’t get it” or “don’t understand”.

Faith Community (*n* = 6/19): Some participants discussed the role of faith and religion in their lives. Faith communities offered an additional layer of psychosocial support: “I have a group of women that we meet once a month through the church… [Child] is on the prayer list for our group, and people will frequently ask me how she’s doing, and how I’m holding up”. Participants also explained how their faith helped get them through acutely stressful experiences. One caregiver remarked, “You just feel the Bible lift you. It’s just incredible because [God] shows you. He’s there. He’s going to be there. And it helps a lot… You know that you’re not alone”.

### 3.3. Theme 3: Making Time for Self-Preservation

Lastly, all participants (*n* = 19/19) described purposefully carving out time for themselves as an essential form of “self-preservation”. Dedicated time for self-care was essential to preventing “breakdown” and maintaining one’s long-term ability to perform in their caregiving role. Participants discussed several strategies allowing them to shift attention inwards.

Little Things (*n* = 14/19): Multiple caregivers discussed intentionally taking time to find pleasure in everyday activities unrelated to their child’s medical care. One parent commented, “You can’t forget the little things that you still enjoy, like you can still put on your favorite band and listen to [them] with your kid”. Participants also made time for interests that contributed to their self-identity, such as hobbies (e.g., one father raced motocross) or community organization participation. Caregivers acknowledged how difficult finding the time for these endeavors could be due to time constraints, planning requirements, and a lack of child-care options. However, participants emphasized the self-preserving aspects of this strategy. One mother explained, “I am starting to find me again. Because for a long, long time, there wasn’t any me… I don’t care if that means just me for an hour here and there… but you definitely need to make time for yourself”.

Emotional Space (*n* = 13/19): Most participants also discussed the importance of finding time to feel and process their emotions. Making an effort to relish positive feelings was described as rejuvenating and healing. For example, one mother described her routine when life becomes overwhelming, “[I] just try to take a moment to sit down and hold [child] in the chair and just cuddle him and take a moment to really appreciate how my boys are going. … Those kinds of things are food to my soul”. Central to creating the time and space necessary for emotional processing was developing the ability to be present in any given moment, slowing down, and stopping thinking about the challenges or tasks at hand.

In contrast, caregivers also explained that indulging negative emotions (e.g., anger), albeit temporarily, could be helpful in moving past frustrations and setbacks. Describing her experiences with a palliative care social worker, one participant stated:

Allowing a person to be where they are at is important, like helping them to understand why they’re feeling that way. [Social worker stating] ‘You’re angry, and this is why you’ve got the right to be’’. … I think it’s important for me to be okay with what I’m feeling at the moment, just making it safe.

## 4. Discussion

In this qualitative study, family caregivers of CMC described the strategies they used to cope with stress related to their caregiving experience. While participants reported a variety of approaches for dealing with stress and challenges, three overarching themes emerged: (1) maintaining a positive mindset, (2) developing and relying on interpersonal support networks, and (3) making time for self-preservation. This study responds to national calls for a better awareness and understanding of CMC family caregivers’ experiences [13,23]. Our findings can inform the development and implementation of services and structural reforms aiming to better support CMC family caregiver well-being [9,10,24]. More specifically, our results highlight potential future directions for psychosocial, clinical, and communication interventions targeting CMC family caregivers’ ability to cope with stress.

### 4.1. Maintaining a Positive Mindset

Our first theme, “maintaining positivity”, aligns with prior findings from the caregiver coping literature, which has shown that a positive mindset is associated with enhanced mental and physical health, quality of life, and care quality, as well as a decreased perceived burden [25,26,27]. More specifically, our subthemes of being hopeful and finding joy, accepting clinical realities, and developing a sense of pride are consistent with research describing hope, prognostic awareness, and “good parent” beliefs among parents of children with serious illnesses [28,29,30,31]. Our study adds to this body of work by demonstrating these subthemes’ interconnectedness and how each represents potential strategies for CMC family caregivers to cultivate a positive mindset, which has important clinical and research implications. For example, CMC clinicians could be more intentional in promoting and maintaining mindset positivity by routinely exploring family caregivers’ hopes and/or good parent beliefs, affirming their aspirations, and praising caregivers’ efforts [20,29,32]. Furthermore, clinician leaders could partner with CMC families to examine how their local health system’s structure and team’s services could be better tailored to nurture caregivers’ hopes and good parent ideals [28]. As only a minority of family caregivers report engagement in similar discussions, future research and educational directions include the development and testing of additional communication frameworks that specifically target CMC family caregiver hope, acceptance, and good parent beliefs. Perhaps more importantly, how such communication strategies can be better incorporated into clinician training and translated into clinical practice must be examined [31,33].

### 4.2. Developing Interpersonal Support Networks

Our participants’ discussions about the importance of robust interpersonal networks and self-preservation are consistent with the existing caregiving literature as well. Social support from a variety of sources (e.g., extended family, clinicians, faith communities) has been repeatedly linked to reduced emotional distress, an enhanced sense of overall well-being and physical health, and a lower perceived burden among multiple caregiver populations [34,35]. Likewise, caregiver engagement in self-care, which refers to practices that promote and maintain one’s health and well-being, are associated with reduced emotional distress and perceived stress, increased satisfaction with the caregiving role, and improved outcomes for care recipients among caregivers of adults with serious illnesses [36,37].

Our findings highlight aspects of these coping strategies which are especially pertinent to family caregivers of CMC. First, almost all participants described how other CMC caregivers served as key pillars in their interpersonal support networks, even if most communication occurred virtually. Fellow caregivers were a source of informational support, such as for practical advice for troubleshooting medical technology or which subspecialists could help with specific issues. They also provided critical forms of emotional support, with caregivers reporting receiving moral support and encouragement during acutely stressful times in our study. Participants also reported that the camaraderie and friendship they felt with fellow CMC caregivers helped mitigate feelings of loneliness and perceived stigma from others. Therefore, CMC clinicians should be knowledgeable about local and national caregiver organizations (e.g., Maternal and Child Health Bureau’s Family-to-Family Health Information Centers) and incorporate this information as part of a child’s comprehensive care plan. This also suggests that the further development and dissemination of programs connecting CMC family caregivers, such as virtual support groups or peer navigator programs, represent high-yield opportunities for investigators and leaders within the complex care field [38,39].

### 4.3. Making Time for Self-Preservation

In discussing self-preservation, participants emphasized that self-care practices must fit within the time constraints, unpredictable schedules, and demanding responsibilities characterizing most CMC caregivers’ experiences. Therefore, clinicians’ attempts to promote self-care should focus on strategies in which caregivers can be self-taught and are of short duration. Our participants’ discussion of enjoying “little things” and/or creating “emotional space” suggests that psychosocial interventions such as mindfulness meditationand/or meaning-making activities might be particularly suitable for CMC caregivers [40,41]. We also highlight that the external factors limiting our caregivers’ ability to engage in self-care provide additional evidence that structural and policy reforms that bolster home health supports and/or formal respite service opportunities are critically needed [42].

A summary of recommendations and resources for CMC clinicians to enhance their support of CMC family caregiver coping is presented in Table 5 [23,28,29,43].

## 5. Limitations

This study was completed at a single institution which may limit the generalizability of our findings. Relatedly, self-selection in choosing to participate may influence the data given the smaller sample size of participants. Although we recruited a racially diverse sample, our participants primarily identified as mothers, which may limit the understanding of the caregiver’s experience from the paternal perspective. Additionally, caregivers who were non-English-speaking were excluded, so the specific coping mechanisms of this population may not be inferred through our study. Lastly, as our study consisted of a one-time interview with caregivers with an average age of 43 years, the feasibility and acceptability of caregivers earlier in their journey utilizing these coping strategies might be low.

## 6. Conclusions

In summary, family caregivers of CMC utilize a multi-faceted set of coping strategies to deal with the stress and challenges inherent to CMC caregiving. Broadly, these strategies focused on maintaining a positive mindset, developing a network of social support, and practicing self-care. We believe our findings can guide ongoing clinical, research, and system-level efforts aiming to enhance our field’s support of CMC family caregivers.

## Figures and Tables

**Table 1:** Demographics information.

Participant Characteristics	
**Caregiver**	***n* (%)**
Age (years)	Mean 43 (SD 6.7) Range: 32–54
Gender: Female	17 (89.5)
Race/Ethnicity - White - Black - Multi-Racial - Hispanic	14 (73.6) 4 (21.2) 1 (5.3) 1 (5.3)
Duration of Caregiving (years)	Mean 10.6 (SD 6.3)
Presence of Other Adults in Household	13 (68.4)
Presence of Other Children in Household	11 (57.9)
Paid Help with Caregiving	11 (57.9)
Child	*n* (%)
Age (years)	Mean 10.8 (SD 6.3) Range: 1–20
Gender: Female	10 (52.6)
Most Common Organ System Affected - Neurologic - Gastrointestinal - Respiratory	19 (100) 19 (100) 16 (82.4)
Medical Technology Use - G-Tube - Mechanical Ventilation - Wheelchair - Home Oxygen	19 (100) 18 (94.7) 7 (36.8) 16 (84.2) 11 (57.9)
Insurance Type Public (Medicaid) Commercial (Employer-based) Public AND Commercial	9 (47.4) 2 (10.5) 8 (42.1)

**Table 2:** Illustrative quotes for Theme 1 subthemes.

Theme 1: Maintain a Positive Mindset	
Hope and Joy	“I’m just normally a very positive person, and that’s a choice I made a long time ago. If you’re positive, positive comes to you. Positive happens to you, and you make everyone around you positive. So, I’ve just always tended toward positive, even when bad things happen. …I know with my way of thinking and being positive… the positive part of me that will carry me through”. One participant describing how she stays mentally tough… “I think, it’s just knowing, ‘Hey, we have a great track record.’ We’ve gotten through this before, and if I’ve gotten through all these times before—I think, gives you more confidence in knowing that you can get through it again”. “But, like I said, when she just gives me that look and I just know—there’s also times—she doesn’t do much purposeful grabbing and stuff like that, but there’s times when she’s doing that stuff, if my hand’s close enough, she’ll take that left hand of hers and she’ll squeeze a finger or two and just hold on to them. And she won’t let go. I’ll move my arm and she just lets hers come with it. But it’s just the fact that she knows I’m there for her and I do feel like she is comforted by that fact”.
Acceptance	One mother on the advice she would give to her younger self, “To not get too caught up in expectations. I just think, ‘[if] we can fix this, if we fix that, then this will be okay’… [it can end up] that this mindset is all you’re doing and all you’re thinking about”. “I do believe this has made me a stronger person. Because you do have to look at some things and you do have to realize that you can’t change this, you can’t change that, and this may happen, that may happen. And you finally figure out and understand that you’re not always going to have a play in the book. Things are going to happen and… I understand that I can’t control this, I can’t control that… Sometimes [it’s] hard to acknowledge that you don’t have that control”. “But I think instead of it being overwhelming—at the beginning it was—but it’s made me appreciate life a lot more in a weird way. Even though I get stressed because, like I told you, you’re always on. But I feel more balanced now. I don’t let the little things bother me. I don’t worry about dusting my windowsills. I used to worry about my house being really clean when people came over. I don’t care. Doesn’t matter. I’m getting through the day. That’s all that matters. And my kids are healthy and doing good. So I think it really helped me just get rid of a lot of petty things—or little things that I used to worry about”.
Pride	“So when [child] does something that maybe someone else deems as little, I’m just like, ‘Oh, my goodness. This is the greatest thing ever.’ All because I know how far she’s come. So that just makes me feel good in that everything I’ve done for her has been worth it. Everything that I continue to do has been worth it and has made a difference in her life. And in my life too”. One participant, reflecting on her child’s progress: “I totally give him a lot of that credit, most of that credit. But then also I know it’s because I’ve been diligent in my care, and so that is rewarding as well”.

**Table 3:** Illustrative quotes for Theme 2 subthemes.

Theme 2: Developing and Relying on an Interpersonal Support Network	
Family and Friends	“We have a lot of family support, so that really helps. Grandma was here yesterday. And then, we have both our parents live close by and help out a lot… But especially if he’s in the hospital, my mom can come sit with him all day, and the nurse can do his medical care, and she can just hold and snuggle [him]. And I can go home and play with my daughter for the day. So that really helps a ton. I don’t know how people without family support systems do any of this”. Speaking about her relationship with her partner, “Just a lot of what I’m dealing with and what we can try to do to improve. We’re a good team. We’re always trying to put our heads together to try to figure out, ‘What can we do to better the situation? How can we have done something different?’ Just because we’ve been doing this for so long… We’re just a good team. We work together and try different things”. “Every day I’m proud of me. Every day I’m proud of Dad. Every day I’m proud of [child]. Like mentioned earlier, we’re a little team over here. It’s like us against the world, because no matter how much you broadcast it or tell people what you go through every day, nobody, besides us three, know the little details of what it takes to be a strong family. So, I would say every day has a little bit of proud moments”.
In the Trenches	“Friendships that I’ve formed that have been really helpful are other special-needs moms who are in the trenches, so to speak, and can understand the day-to-day struggles and the frustrations and joy that can go along with being a mom to a special kiddo. And those friendships are more so for venting, for emotional support, or encouragement. If you’re feeling down, then they can say, ‘Okay. You’re not alone. We’ve been there. You’re okay. You can make it. You’re a great mom.’ That kind of a thing”. “There’s a Rett syndrome community on Facebook that I will go to. And I do not post anything there, but I kind of read the questions that are asked, and read the answers, and stuff like that. So I’m learning more things and seeing other people’s opinions on things or how they deal with things”. “Just a place to vent because some of my friends don’t have kids with disabilities, so they don’t get it… Because I don’t know if they always understand what I’m talking—other friends without kids with disabilities don’t understand what I’m talking about or understand how some things are important”.
Faith Community	“Well, I believe in prayer a lot. So, I do take time out for prayer every day. So, I find my strength in God. I go to church once a week. And when we are at the hospital, I’ll ask our priest here at our church to please pray for [child], and I know that he does. And he’ll check in on her through text and ask how she’s doing. And then, there’s the chaplain, the priest that visits Children’s Hospital. So he’ll come in, and offer prayers, and whatnot. And then, I have a group of women that we meet once a month through the church”.

**Table 4:** Illustrative quotes for Theme 3 subthemes.

Theme 3: Making Time for Self-Preservation	
Little Things	“Getting away from it for a little while can be helpful. The thing I try to do is every now and then go somewhere just me, whether it’s going into town to pick up meds and I just take a little extra time to shop or go out to eat or getting with a friend or family member to take her out to eat and just try to enjoy some time as [Participant] and not as Mom”. “I mean, you do have to have your own time. I mean, granted, when she’s sleeping and stuff, I read. Well, I do more of that now than I did. I read. Because I love to read, so I read”. “I do find that people ask when do you get a break? When do you take care of yourself? Are you taking care of yourself? And sometimes there’s—I can’t take care of myself because I’m constantly having to take care of my child”.
Emotional Space	“So, I found for me that I still have anxiety and stuff over his future or the present, but I’ve learned that for the more scary stuff, I end up just kind of having my fall-apart moments afterwards. I just kind of take it in stride as it’s happening, and then I fall apart later”. … “whenever I get home, that’s whenever, I guess, because I know that [I] got through that situation, and so then I feel like I kind of deflate or crash because it’s over”. “I think a lot of the frustration often leads to a lot of the overthinking. And I’ve found that—I know that when I’m getting frustrated, I know that I need to take a little more time for myself to kind of wind down and kind of self-regulate a little more and be a little more realistic about my expectations and knowing what I can control and what I can’t”.

**Table 5:** Clinician recommendations and resources to support CMC family caregiver coping.

Theme	Clinician Recommendations and Resources
Maintaining a Positive Mindset	Explore and affirm family caregivers’ hopes and/or good parent beliefs [28,29] ○ “I am hoping for that too. Can I ask what else you are hoping for?” ○ “Other family caregivers have explained they have a sense of what they need to do be a good parent on their own terms. Do you have any feelings like that?” Encourage and praise caregivers’ efforts [29] ○ “Your love and devotion for your child is inspiring”. ○ “How else can we support you in taking the best possible care for your child?” Center and celebrate moments of joy [43] ○ “Could you tell me a bit about what brings your child joy?” ○ “Would you mind sharing a meaningful photo or video of you and your child?”
Developing Interpersonal Support Networks	National Family Organizations and Resources ○ Maternal and Child Health Bureau’s *Blueprint for Change* [15] and Family-to-Family Health Information Centers (https://mchb.hrsa.gov/programs-impact/programs/f2f-health-information-centers Accessed on 22 August 2024) ○ Lucille Packard Foundation Foundation’s Program for Children and Youth with Special Health Care Needs (https://lpfch.org/program-for-children-and-youth-with-special-health-care-needs/ Accessed on 22 August 2024) ○ Courageous Parents Network (https://courageousparentsnetwork.org/ Accessed on 22 August 2024) ○ Family Voices (https://familyvoices.org/ Accessed on 22 August 2024) ○ Collaborative Improvement and Innovation Network (CoIIN) (https://ciswh.org/project/coiin-cmc Accessed on 22 August 2024)
Making Time for Self-Preservation	American Board of Pediatrics’ *The Roadmap Project* (https://www.roadmapforemotionalhealth.org/ Accessed on 22 August 2024) NIH *Your Healthiest Self: Wellness Toolkits* (https://www.nih.gov/health-information/your-healthiest-self-wellness-toolkits Accessed on 22 August 2024) Not-For-Profit Meditation Apps: *Smiling Mind* and *MindShift*^TM^

## Data Availability

The original contributions presented in this study are included in the article, further inquiries can be directed to the corresponding author.
